# Effect of oil-enriched tomato powder and simvastatin on yolk cholesterol and color in laying hens

**DOI:** 10.5455/javar.2025.l999

**Published:** 2025-12-25

**Authors:** Ulvi Fitri Handayani, Maria Endo Mahata

**Affiliations:** 1 Research Center for Animal Husbandry, National Research and Innovation Agency, Bogor, Indonesia; 2 Faculty of Animal Science, Universitas Andalas, Padang, West Sumatera, Indonesia

**Keywords:** Cholesterol, Lohmann-Brown, Lycopene, Tomato, Yolk color

## Abstract

**Objective:**

This study was conducted to investigate the influence of varying levels of oil-enriched tomato powder (OTP) and simvastatin supplementation on the performance and egg quality of laying hens.

**Materials and Methods:**

Two hundred 32-week-old Lohmann–Brown hens were utilized in this study and divided into five groups: T1 = control diet; T2 = control diet + 0.03% simvastatin; T3 = 6% OTP in diet; T4 = 12% OTP in diet; and T5 = 18% OTP in diet.

**Results:**

The treatments with different levels of OTP and simvastatin had no significant effect on the performance of laying hens (*p* > 0.05), including egg weight, egg mass, hen-day egg production (HDEP), feed intake, and feed conversion ratio. However, dietary OTP at T5 significantly enhanced the yolk color index (*p* < 0.05) compared with treatments T1, T2, T3, and T4. Dietary OTP in T5 and supplementation of simvastatin at T2 significantly reduced (*p* < 0.05) cholesterol level in egg yolk, with the lowest cholesterol level produced by treatment T2.

**Conclusion:**

Dietary inclusion of 18% OTP in the diet improves yolk color index and reduces cholesterol without affecting laying hen performance, though simvastatin is more effective in cholesterol reduction.

## Introduction

Tomatoes are an abundant agricultural crop in Indonesia. In 2023, Indonesia produced 1,143,788 tons of tomatoes [1], but approximately 10% of these were discarded as waste. They are from fresh tomatoes that were not harvested on the seventh or eighth day of harvest or from fresh tomatoes that will be marketed [2]. Tomato powder contains 11.92% crude fiber (CF), 10.88% crude protein (CP), 3.85% fat, 0.26% calcium (Ca), 0.69% phosphorus (P), and 1,596 Kcal/kg of metabolic energy [3]. Tomatoes are rich in phytochemical compounds such as lycopene [4]. Lycopene is a well-known antioxidant that helps reduce cholesterol [5,6] and enhances the yolk color index [5,7].

The pigmentation of the egg yolk is an important factor to consider in the laying hen industry. Most people prefer darker yolk colors [8]. This relates to the common perception that a darker yolk is healthier, because a dark color is thought to indicate a higher nutritional value and also a more delicious flavor [8]. Furthermore, eggs are known to be a food product high in cholesterol, which can have potentially harmful effects if consumed excessively [9]. For example, egg consumption is linked to an increased risk of cardiovascular disease (CVD) [10]. However, a meta-analysis reported by Krittanawong et al. [11] showed that egg consumption is not connected with CVD. Eggs with low cholesterol content are a beneficial solution for producing healthy food products. Consequently, using tomato waste as a lycopene source in laying hen feed presents an opportunity to produce healthy eggs with less cholesterol content.

Lycopene reduces cholesterol through a mechanism similar to that of statins. Simvastatin is a statin medication, a 3-hydroxy-3-methylglutaryl coenzyme A (HMG-CoA) reductase inhibitor, the key enzyme involved in cholesterol biosynthesis [12]. Nevertheless, the bioavailability of lycopene from natural sources in the digestive tract is relatively low. Only about 7%-10% of lycopene is absorbed through the intestinal tract [13]. Factors such as the presence of fat [14], the configuration of lycopene [13], and the structure of the plant cell wall [15] significantly influence lycopene absorption. According to previous research, processing tomatoes by heating can increase lycopene content. The steaming method at 98°C could maintain the ash content, and the heating duration of 12 min is ideal for increasing lycopene [16]. Adding 0.5% coconut oil to steamed tomato powder for 12 min was found to be the optimal type and dose of oil for increasing nitrogen retention, improving the digestibility of CF, and enhancing lycopene retention in broiler chickens [3]. However, no studies have evaluated steamed tomato powder enriched with coconut oil in laying hens. In summary, this study aimed to investigate the impact of varying levels of oil-enriched tomato powder (OTP) and simvastatin on the performance and egg quality of laying hens.

## Materials and Methods

### Ethical approval

The use of animals and veterinary drugs in this study complied with the institutional animal care and ethics guidelines and was approved by the Animal Ethical Committee of Universitas Andalas, Padang, Indonesia, Faculty of Medicine (Approval No. 574/KEP/FK/2019), registered on 04 November 2019.

### Preparation of OTP

The OTP was produced in accordance with the methodology established by Handayani et al. [3]. Tomato waste at the optimal ripening stage was collected from an agricultural area in Alahan Panjang District, West Sumatera Province of Indonesia. Subsequently, these tomatoes were steamed at 98°C for 12 min and dried for 3 days at 60°C. The tomatoes were then ground and blended with 0.5% coconut oil. The proximate analysis was performed according to the AOAC method, and the amino acid profile was assessed using High-Performance Liquid Chromatography at the integrated laboratory of IPB University (Table 1).

**Table 1.** Nutrient content and amino acids of OTP meal.

Nutrients ^*^	Value
CP (%)	10.88
Fat (%)	3.85
CF (%)	11.92
Ca (%)	0.26
Ps (%)	0.69
Metabolizable energy (Kcal/kg)	1,596.00
Lycopene (mg/kg)	52.10
Amino acid composition (ppm)	
Alanine	0.97
Arginine	1.67
Aspartic acid	1.62
Glutamate	2.55
Glysine	0.85
Histidine	0.59
Ileusin	0.80
Leusine	1.27
Lysine	0.83
Methionine	0.25
Phenylalanine	0.71
Serine	0.76
Threonine	0.75
Tyrosine	0.49
Valine	0.89

*Analyse data.

### Experimental design, animal, diet, and housing

Two hundred female Lohmann-Brown, aged 32 weeks and weighing 1,600–1,800 gm, were sourced from a commercial rearing farm in Pariaman District, West Sumatera Province, Indonesia. The hens were housed under consistent husbandry conditions, including a 12-h light and 12-h dark cycle, and had unlimited access to water and feed. They were individually housed in cages (length, 40 cm; width, 30 cm; height, 30 cm). A completely randomized design was employed in the experiment, consisting of five dietary groups, each with four replications (*n* = 10), following a 1-week adaptation period. The interventions in the experiment consisted of T1 = control diet; T2 = control diet + 0.03% simvastatin; T3 = 6% OTP in diet; T4 = 12% OTP in diet; and T5 = 18% OTP in diet. The feeding trial was initiated on 33-week-old layers and continued until 39 weeks of age (6 weeks). The diets were formulated based on the National Research Council [17] guidelines for poultry; the ration was based on isoprotein and isoenergetic principles. The dietary nutrient composition is shown in Table 2.

**Table 2.** Dietary ingredients and nutrient composition of the diet.

Items	Content				
	Control (T1)	Control + 0.03% simvastatin ^1^(T2)	6% OTP in diet (T3)	12% OTP in diet (T4)	18% OTP in diet (T5)
Ingredients, %					
Concentrate^2^	26.30	26.30	26.30	26.30	26.30
Maize grain	47.40	47.40	47.40	47.40	47.40
Rice bran	21.10	21.10	15.10	9.10	3.10
OTP	0.00	0.00	6.00	12.00	18.00
Mineral mix^3^	0.50	0.50	0.50	0.50	0.50
Dolomite^4^	4.70	4.70	4.70	4.70	4.70
Total	100.00	100.00	100.00	100.00	100.00
Nutrient composition					
Metabolizable energy (Kcal/kg)	2,631	2,631	2,629	2,627	2,625
CP(%)	16.67	16.67	16.60	16.54	16.47
CF (%)	5.04	5.04	4.80	4.55	4.31
Fat (%)	4.88	4.88	4.47	3.78	3.65
Ca (%)	3.32	3.32	3.32	3.31	3.31
P (%)	0.40	0.40	0.43	0.45	0.48
Methionine (%)	0.37	0.37	0.37	0.37	0.36
Lysine (%)	0.60	0.60	0.61	0.63	0.64
Lycopene (mg/kg)	0.00	0.00	31.26	62.52	93.78

^1^simvastatin using commercially product by Kimia farma, Indonesia containing 20% simvastatin); ^2^concentrate using commercially product by New Hope Indonesia; ^3^ mineral mixes using commercially sindomix-binder (premix) product by Otasindo, Indonesia;^ 4^dolomit using commercially product by Usali, Indonesia; OTP was processed from steaming tomatoes waste mix with 0.5% coconut oil.

### Laying hens' performance

The parameters of laying hens’ performance consist of feed intake (FI), hen-day egg production (HDEP), feed conversion rate (FCR), egg mass, and egg weight. FI was recorded every week. Egg mass and egg weight were recorded every day for each treatment. The FI, HDEP, FCR, egg mass, and egg weight were calculated as follows:


$$\mathrm{FI}(\mathrm{gm}/\mathrm{hen}/\mathrm{day})=\frac{\mathrm{Feed}\;\mathrm{consumed}(\mathrm{gm})}{\mathrm{The}\;\mathrm{number}\;\mathrm{of}\;\mathrm{bird}\;\times \;\mathrm{Days}}$$

$$\mathrm{HDEP}\mathrm{(\%)}=\frac{\mathrm{Tota}l\;n\mathrm{umber}\;\mathrm{of}\;\mathrm{eggs}}{\mathrm{The}\;\mathrm{number}\;\mathrm{of}\;\mathrm{bird}\;\mathrm{present}}\times 100$$

$$\mathrm{FCR}=\frac{\mathrm{Total}\;\mathrm{FI}(\mathrm{gm})}{\mathrm{Total}\;\mathrm{egg}\;\mathrm{mass}(\mathrm{gm})}$$

Egg mass (gm/hen/day) = Egg production (%) × average egg weight (gm)


$$\mathrm{Egg}\;\mathrm{weight}(\mathrm{gm}/\mathrm{egg})=\frac{\mathrm{Total}\;\mathrm{weight}\;\mathrm{of}\;\mathrm{egg}(\mathrm{gm})}{\mathrm{The}\;\mathrm{number}\;\mathrm{of}\;\mathrm{egg}(\mathrm{egg})}$$

### Egg quality analysis

During the 7-week feeding trial, fresh eggs were collected on the 48th and 49th days. Two eggs from each replicate (*n* = 8 per group) were collected to evaluate egg quality. The parameters used to assess egg quality included eggshell thickness and strength, egg length, egg width, Haugh unit (HU), albumin height, egg yolk color index, egg yolk weight, egg yolk fat, and egg yolk cholesterol. Eggshell thickness, egg length, and width are measured by a millimeter screw. Eggshell strength was measured with the Egg Force Reader (Orca Food Technology Ltd.). Egg yolk color indexes were measured with the Roche Yolk Color Fan.

The HU was calculated as follows:

HU = 100 Log (H+7.57- 1.7 × W^0.37^)

where W = egg weight (gm), H = albumin height, and HU = Haugh Unit.

The egg yolk was separated from the albumin and dried in the oven at 60°C for 24 h. The dry egg yolks were ground, and subsequently, egg yolk fat was measured utilizing the AOAC approach (AOAC 1990).

### Analysis of cholesterol levels in egg yolk

The cholesterol concentration in the egg yolks was analyzed using a modified Liebermann-Burchard Reaction method [18].

### Preparation of cholesterol standard solution

The cholesterol standard was weighed up to 300 mg and then dissolved in chloroform in a 25 ml volumetric flask to the calibration mark. A standard solution was then produced with a concentration of 12,000 ppm (solution A). Subsequently, dilutions were made with 1.0, 0.8, 0.6, 0.4, and 0.2 mg/ml in 5 ml standard solution. Two ml of acetic anhydride and sulfuric acid in a 30:1 v/v ratio were added to each dilution. The dilutions were then placed in a dark room for 5 min until the solutions turned green. Absorbance was calculated with a Shimadzu UV-1800 spectrophotometer at a wavelength (*λ*) of 680 nm, which was used to generate the standard curve equation.

### Egg yolk extraction

A sample of as much as 0.5 gm (A) was placed in a centrifuge tube containing 5 ml of a solution of acetone: ethanol (1:1 v/v). The tube was then tightly closed and vortexed for 1 min, allowed to stand for 30 min afterwards, and then heated in a water bath until boiling. Subsequently, the tube was removed and allowed to cool to room temperature before being centrifuged at 1,500 rpm for 15 min. The formed supernatant (clear part) was placed in a test tube and evaporated in a water bath until dry, creating a paste (residue). The obtained residue was then dissolved in 3 ml of chloroform. It was then diluted to the desired dilution factor. Then, 2 ml of solution (A) was added to 2 ml of acetic anhydride: sulfuric acid (30:1 v/v), placed in a dark room for 5 min until it turned green, and the absorbance was measured with a spectrophotometer at 680 nm. The obtained absorbance in ppm is entered into the obtained regression formula, which is then converted to mg/100 gm.

### Statistical analysis

Data analysis was performed using analysis of variance to determine the significance of the treatments' effect. Differences in treatments were determined with Tukey’s HSD test. Data are shown as means ± standard deviation (SD), and statistical significance was considered at *p* < 0.05. All statistical analyses were performed using JASP v. 0.19.2 software.

## Results

### Performance of laying hens

**Figure 1.**
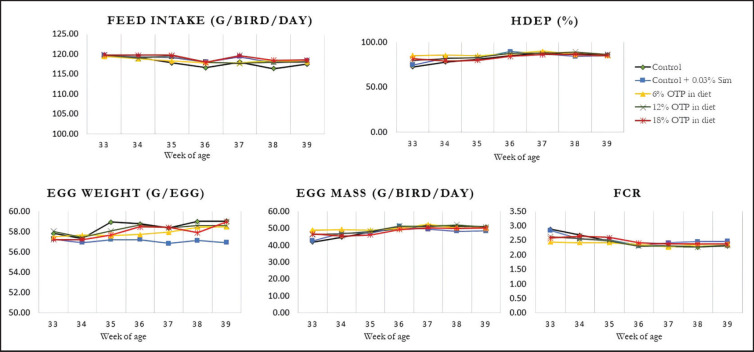
The transition of FI, HDEP, egg weight, egg mass, and FCR.

The dietary treatments showed a relatively consistent pattern in FI, HDEP, FCR, egg mass, and egg weight throughout the 7-week feeding trial (Fig. 1). FI, HDEP, FCR, egg mass, and egg weight were not significantly different among the groups (*p* > 0.05) (Table 3). These results indicate that the addition of OTP at various levels and simvastatin do not affect the performance of laying hens.

**Table 3.** Effect of dietary treatments on performance of laying Hens1.

Treatments	Feed Intake (gm/hen/day)	HDEP (%)	Egg Weight (gm/egg)	Egg Mass (gm/hen/day)	FCR
Control (T1)	117.89 ± 1.14	82.57 ± 1.16	58.47 ± 1.10	48.30 ± 0.91	2.46 ± 0.06
Control + 0.03% Sim (T2)	118.84 ± 0.88	83.65 ± 3.61	57.07 ± 0.53	47.69 ± 2.07	2.51 ± 0.12
6% OTP in diet (T3)	118.40 ± 1.09	86.17 ± 3.73	57.90 ± 1.57	49.94 ± 2.97	2.38 ± 0.13
12% OTP in diet (T4)	118.57 ± 1.92	84.95 ± 3.47	58.25 ± 0.93	49.49 ± 1.65	2.41 ± 0.13
18% OTP in diet (T5)	119.11 ± 0.71	83.21 ± 3.85	57.98 ± 0.78	48.27 ± 1.90	2.48 ± 0.10
*p*-value	0.804	0.569	0.419	0.507	0.484

^1^Data: mean ± SD; HDEP, Hen Day Egg Production; FCR, Feed Conversion Rate; OTP, oil-enriched tomato powder; Sim, Simvastatin; Data analyzed using one-way ANOVA followed by Tukey’s test; The analysis was performed with the mean of the performance of all individuals in each group over 7 wk (*n* = 40 each group); SEM: Standard error of the mean.

### Egg quality parameters

The effect of treatment on eggshell thickness and strength, egg length, egg width, HU, albumin high, and yolk parameter (weight, color, fat, and cholesterol content) are presented in Table 4. Egg length and width, eggshell thickness and strength, HU, yolk weight, and yolk fat were not significantly different among the groups (*p* > 0.05). A significant difference was observed in the yolk color index (*p* < 0.05), with a proportional increase as the OTP supplementation level increased. Yolk color index in the treatment T5 (18% OTP) is higher (*p* < 0.05) than in all treatment groups. These results indicate that the lycopene content in OTP effectively enhanced egg yolk color intensity. The T2 (Simvastatin) group exhibited the lowest cholesterol content (603.58 mg/100 gm), which was significantly lower (*p* < 0.05) than all other treatments. Nevertheless, the treatments T4 (12% OTP) and T5 (18% OTP) also showed a reduction in cholesterol levels in egg yolk, although treatment T3 showed no significant difference (*p* > 0.05) from the control treatment (T1). The most effective method for reducing cholesterol in egg yolks is the administration of 0.03% simvastatin (T2). However, a diet containing 18% OTP is the most appropriate treatment for enhancing the egg yolks’ pigmentation.

**Table 4.** Effect of dietary treatments on egg quality1.

Item	Control (T1)	Control + 0.03% Sim (T2)	6% OTP in diet (T3)	12% OTP in diet (T4)	18% OTP in diet (T5)	*p*-value
Egg length (cm)	5.61 ± 0.12	5.55 ± 0.04	5.6 ± 0.08	5.57 ± 0.08	5.62 ± 0.06	0.765
Egg width (cm)	4.39 ± 0.07	4.23 ± 0.18	4.36 ± 0.09	4.33 ± 0.11	4.37 ± 0.08	0.305
Eggshell thickness (mm)	0.38 ± 0.02	0.39 ± 0.01	0.38 ± 0.02	0.37 ± 0.01	0.36 ± 0.01	0.254
Eggshell strength (kg/cm^3^)	4.93 ± 0.1	4.79 ± 0.19	4.90 ± 0.2	4.66 ± 0.19	4.72 ± 0.17	0.194
Albumin height (mm)	9.86 ± 0.33	10.13 ± 0.35	10.15 ± 0.65	9.67 ± 0.89	9.97 ± 0.5	0.759
HU	98.08 ± 1.2	99.93 ± 1.18	99.49 ± 2.97	97.26 ± 3.76	99.02 ± 1.24	0.569
Egg yolk weight (gm)	14.03 ± 1.01	14.43 ± 0.98	14.61 ± 0.28	14.46 ± 0.75	13.69 ± 0.61	0.461
Egg yolk fat (%)*	27.64 ± 1.97	26.39 ± 1.79	28.31 ± 0.84	27.50 ± 1.21	27.20 ± 0.94	0.455
Egg yolk color index	9.64^bc^± 0.35	9.33^c^± 0.38	9.95^bc^± 0.59	10.58^ab^± 0.41	11.06^a^± 0.39	< 0. 001
Egg yolk cholesterol* (mg/100 gm)	1,113.64^a^± 60.10	603.58^c^± 68.06	1,113.77^a^± 34.03	956.41^b^± 44.22	866.99^b^± 64.03	< 0. 001

^1^Data: mean ± SD; Sim, Simvastatin; Data analyzed using one-way ANOVA followed by Tukey’s test; The analysis was performed with the mean of the performance of all individuals in each group over 7 wk (*n* = 40 each group); SEM: Standard error of the mean; ^a,b,c,^ Mean values within arow with different superscripts letters represent the statistical differences; *p* < 0.05;^*^% in fresh matter.

## Discussion

### Performance of laying hens

Improving laying hen performance is critical to enhance economic efficiency [19]. The results showed that the addition of OTP in the diet, up to 18%, did not compromise the palatability of the ration. Additionally, OTP at this level could substitute rice bran up to 85.3% in laying hen diets (Table 2). These results suggest that OTP is a suitable alternative feed ingredient that can be effectively integrated into laying hen diets without negatively affecting performance. Previous research investigated the impact of the supplementation of tomato waste on the performance of laying hens. Dietary tomato waste (12%-19%) did not significantly affect the feed consumption of laying hens [20–22. Additionally, the inclusion of up to 15% tomato waste showed no improvement in the FCR of laying hens [23].

Different studies stated that the inclusion of 10% tomato waste increased egg mass [24] and egg production [25], while 15% resulted in a decrease in both production and egg mass [25]. Dietary tomato waste at 20% resulted in an increase in dry matter intake without significantly affecting body weight or FCR [26]. In another study, it was proven that dietary tomato waste, up to 7.5%, decreased FI [27]. A recent meta-analysis investigated the influence of various types of tomato waste on feedstuffs in laying hen diets. They reported that dietary tomato waste exhibited a quadratic correlation with egg production and egg mass, while FI increased in a linear pattern [5]. This variation may be attributed to differences in the source of tomato waste and its processing, which is a critical factor in the nutrient content of feed. The nutrient content of tomato waste from whole tomatoes contains 25.19% CF, 10.73% CP, and 2.18% crude fat [28]. In comparison, tomato pulp has 36.16% CF, 19.17% CP, and 10.3% crude fat [29]. Safamehr et al. [30] explained that tomato pomace (tomato by-product from processed tomatoes) contains 29.79% CF, 19.68% CP, and 8.1% crude fat [30].

This present research shows that the average egg weight ranged from 57.07 to 58.47 gm/egg. This result is similar to Habanabashaka et al. [31], who reported that the average egg weight of laying hens aged 36–44 weeks fed tomato waste ranged from 58.10 to 58.40 gm. However, this result differs from the findings of An et al. [32], who reported that the average egg weight of Hy-Line Brown hens aged 38–42 weeks given tomato pulp ranged from 63.1 to 65.9 gm. The FCR obtained in the current study was similar to the FCR of laying hens reported by Salajegheh et al. [22], which ranged between 2.25 and 2.42. Nevertheless, studies on simvastatin supplementation in laying hens are still limited. Kim et al. [33] reported that simvastatin supplementation at 0.06% did not affect egg weight in laying hens. This finding indicates that the administration of 0.03%simvastatin in laying hens' diet does not interfere with egg formation in the reproductive tract.

### Egg quality parameters

The eggshell thickness and strength showed no significant alterations due to the treatments. These findings are consistent with previous studies, which reported no significant effect on eggshell thickness and strength when using tomato waste in laying hens’ diets [22,33,31,34]. From this investigation, dietary OTP and supplementation with 0.03% simvastatin in the ration did not have a significant influence on the internal parameters of egg quality (HU, high albumin, egg yolk weight, and egg yolk fat). This result, similar to the previous research, reported no effect of tomato waste inclusion on HU [20,22,23,31,34], egg yolk weight [31], and egg yolk fat [5]. Thus, the nutrients in the diet, particularly Ca, P, magnesium, and protein, are effectively digested and absorbed by laying hens. Consequently, the nutritional requirements of the hens are adequately met without compromising the quality of the eggs.

Egg yolk color is key to the quality of an egg. The preference for egg yolks with a higher color index was very popular in most countries, reflecting consumer demand for high-quality eggs [8]. In the present research, dietary inclusion of OTP enhanced the yolk color index. The coloration effect is primarily due to the lycopene content in OTP, which functions as a bioactive carotenoid pigment with potent coloring properties. This result is consistent with previous research, which found that lycopene from tomatoes can improve the yolk color index [35]. The yolk's color is attributed to carotenoid compounds present in the ration [36]. The color index of egg yolk observed ranged from 9.33 (for the control treatment) to 11.06 (dietary 18% OTP), which aligns closely with findings of previous studies. The yolk color index in the present meta-analysis ranged from 1.58 to 14.53 [5].

Previous studies have reported varying effects of tomato waste supplementation on egg yolk color. Adding 12% tomato pulp and 10% tomato pomace was found to improve yolk color in laying hens. However, the addition of 13% tomato waste meal and 15% tomato waste, combined with an enzyme, did not affect the yolk color. In contrast, the yolk color increases when tomato waste is added at levels of 17% and 19% [22]. In the present study, supplementation with OTP up to 12% has not increased yolk color compared to the control treatment. The addition of 18% OTP increased yolk color. Although steaming and coconut oil enrichment effectively increase lycopene absorption in chicken [3], the type of tomato waste is a crucial factor in determining the dosage of tomato waste as a feedstuff for laying hens.

Eggs are a well-balanced food, containing high-quality protein, vitamins, and minerals important to human health. Despite their nutritional benefits, the elevated cholesterol level in eggs has sparked ongoing debate regarding the potential link between dietary cholesterol and cardiovascular risk. Therefore, studies on feed manipulation to reduce cholesterol levels are of great interest. The administered tomato waste and medicine, such as simvastatin, demonstrated their potential to minimize egg yolk cholesterol. Both lycopene and simvastatin reduce cholesterol production in the body. Laying hens meet their cholesterol requirements through endogenous synthesis of 300 mg/day in the liver and ovaries. However, cholesterol produced by the ovary is rarely transported to oocytes and contributes very little to egg cholesterol [33]. The hepatic synthesis of cholesterol in laying hens is subsequently transported via very low-density lipoprotein (VLDL) particles, where it is incorporated into the developing egg yolk [36]. Vitellogenesis receptors in oocytes then internalize VLDL particles, allowing for follicular development and deposition into egg yolk [36]. As a result, limiting liver cholesterol biosynthesis causes a significant reduction in egg cholesterol levels [33].

Some previous research has reported that 9% [31] and 12% [37] dietary tomato waste for laying hens reduced the egg yolk cholesterol level and improved egg yolk color. Furthermore, a meta-analysis found that administering tomato waste had a positive effect on reducing cholesterol content in egg yolks from laying hens. However, the effect did not reach statistical significance [5]. Meanwhile, other literature reports that dietary levels of 10% and 12%, respectively, have not reduced egg yolk cholesterol [25,30]. The reduction in cholesterol levels with OTP administration became evident at a dietary 12%, resulting in a 14.12% decrease. Meanwhile, inclusion of 18% OTP in the diet led to a more significant reduction of 22.15%. However, the greatest reduction in cholesterol levels in egg yolk was observed with the supplementation of 0.03% simvastatin. Supplementation with 0.03% simvastatin decreased the egg cholesterol level by as much as 45.80% from the control treatment. Similar results were also observed by Wang et al. [38], who found that the addition of 150 mg of HMG-CoA reductase inhibitors per kg of diet in laying hens can effectively minimize cholesterol in the serum, liver, pectoral muscles, and egg yolk. According to Elkin [39], research on reducing cholesterol levels in chicken eggs through the administration of pharmacological compounds such as statins has not been well developed. The limited progress of this research is attributed to concerns regarding drug residues, high costs, and regulatory restrictions on the use of pharmacological compounds in poultry production. An investigation by Elkin et al. [40] also demonstrated the presence of drug residues in the egg yolks of hens supplemented with 0.06% atorvastatin. However, our research did not analyze the statin residues in eggs. The use of simvastatin at the applied dietary level (0.03%) is considered safe for poultry products, as this dose is lower than the level reported by Elkin et al. [40].

The findings highlight that simvastatin was more effective than OTP in lowering cholesterol levels in egg yolk. However, despite its efficacy, the application of simvastatin in laying hens is impractical due to potential concerns regarding drug residues in eggs, which pose food safety and regulatory challenges. Therefore, using alternative natural compounds, such as lycopene from tomato waste, is preferable because it provides similar cholesterol-lowering benefits without the associated risk of residue.

## Conclusion

In conclusion, agricultural wastes such as tomato waste, which contain compounds rich in lycopene, may be useful sources of antioxidants and coloring agents as feed ingredients. In summary, the results indicate that including up to 18% OTP in the diet enhanced egg yolk color and lowered egg yolk cholesterol without impacting overall performance. However, supplementation with 18% OTP was still less effective in reducing cholesterol levels compared to the diet containing simvastatin. In further studies, the bioprospecting of agroindustrial by-products is expected to gain increased attention and interest as a means of enhancing production performance, health, and especially product quality in poultry.

## List of abbreviations

ANOVA, analysis of variance; Ca, calcium; CF, crude fiber; CP, crude protein; CVD, cardiovascular disease; FCR, feed conversion ratio; FI, feed intake; HDEP, hen-day egg production; HMG-CoA, 3-hydroxy-3-methylglutaryl coenzyme A; HU, Haugh unit; OTP, oil-enriched tomato powder; P, phosphorus; SD, standard deviation; min, minutes; h, hour; ml, milliliter; kg, kilograms; gm, grams; mg/day, milligrams per day.

## References

[1] Central Bureau of Statistics (2024). Vegetable crop production. https://www.bps.go.id/id/statistics-table/2/NjEjMg==/produksi-tanaman-sayuran.html.

[2] Handayani UF, Wizna W, Suliansyah I, Rizal Y, Mahata ME (2023). Effects of processed tomato (*Lycopersicon esculentum*) wastes in the diet on the serum lipid profile of laying hens. Adv Anim Vet Sci.

[3] Handayani UF, Wizna W, Suliansyah I, Rizal Y, Mahata ME (2019). The evaluation of dietary addition of palm and coconut oils in steaming tomato (*Lycopersicon esculentum*) waste powder on digestibility of crude fiber and retention of lycopene and nitrogen in broiler chickens. J World Poult Res.

[4] Varzaru I, Untea A.E, Panaite T, Olteanu M (2021). Effect of dietary phytochemicals from tomato peels and rosehip meal on the lipid peroxidation of eggs from laying hens. Archiv Für Tierernährung.

[5] Handayani UF, Sofyan A, Lestari D, Sholikin MM, Wulandari W, Harahap MA, et al. (2023). Dietary supplementation with tomato waste to improve performance and egg quality of laying hens: a meta-analysis. J Anim Feed Sci.

[6] Orhan C, Kucuk O, Sahin N, Tuzcu M, Sahin K (2021). Lycopene supplementation does not change productive performance but lowers egg yolk cholesterol and gene expression of some cholesterol-related proteins in laying hens. Br Poult Sci.

[7] Shevchenko LV, Iakubchak OM, Davydovych VA, Honchar VV, Ciorga M, Hartung J, et al. (2021). Influence of lycopene and astaxanthin in feed on metabolic parameters of laying hens, yolk color of eggs and their content of carotenoids and vitamin A when stored under refrigerated conditions. Pol J Vet Sci.

[8] Altmann BA, Trinks A, Mörlein D (2023). Consumer preferences for the color of unprocessed animal foods. J Food Sci.

[9] Antoni R (2023). Dietary saturated fat and cholesterol: cracking the myths around eggs and cardiovascular disease. J Nutr Sci.

[10] Ruggiero E, Di Castelnuovo A, Costanzo S, Persichillo M, De Curtis A, Cerletti C, et al. (2021). Egg consumption and risk of all-cause and cause-specific mortality in an Italian adult population. Eur J Nutr.

[11] Krittanawong C, Narasimhan B, Wang Z, Virk HUH, Farrell AM, Zhang H, et al. (2021). Association between egg consumption and risk of cardiovascular outcomes: a systematic review and meta-analysis. Am J Med.

[12] Handayani UF, Suliansyah I, Rizal Y, Mahata ME (2018). Effect of heating method on lycopene, dry matter and nutrient content of tomato (*Lycopersicon esculentum*) waste as laying hen feed. Int J Poult Sci.

[13] Kumari A (2018). Chapter 7 - cholesterol synthesis. Sweet Biochem.

[14] Wu H, Wu Y, Cui Z, Hu L (2024). Nutraceutical delivery systems to improve the bioaccessibility and bioavailability of lycopene: a review. Crit Rev Food Sci Nutr.

[15] Arballo J, Amengual J, Erdman JW (2021). Lycopene: a critical review of digestion, absorption, metabolism, and excretion. Antioxidants.

[16] Cooperstone JL, Ralston RA, Riedl KM, Haufe TC, Ralf M, King SA, et al. (2015). Enhanced bioavailability of lycopene when consumed as cis-isomers from tangerine compared. Mol Nutr Food Res.

[17] NRC (1994). Nutrient requirements of poultry. National Academies Press.

[18] Adu JK, Amengor CDK, Kabiri N, Orman E, Patamia SAG, Okrah BK (2019). Validation of a simple and robust Liebermann–Burchard colorimetric method for the assay of cholesterol in selected milk products in Ghana. Int J Food Sci.

[19] Dai D, Wu S G, Zhang H J, Qi G H, Wang J (2020). Dynamic alterations in early intestinal development, microbiota and metabolome induced by in ovo feeding of L-arginine in a layer chick model. J Anim Sci Biotechnol.

[20] Mierlita D, Daraban S, Teușdea AC, Stanciu AS (2024). Effect of dietary cold-pressed hempseed cake supplemented with tomato waste on laying hen performance and egg yolk lipid profile and antioxidant status before and after storage. Animals.

[21] Kemer YA, Mengistu U, Shalu K, Negasi A (2022). Effect of tomato waste meal supplementation on laying performance and egg quality of white leghorn chickens. Livest Res Rural Dev.

[22] Salajegheh MH, Ghazi S, Mahdavi R, Mozafar O (2012). Effects of different levels of dried tomato pomace on performance, egg quality and serum metabolites of laying hens. Afr J Biotechnol.

[23] Tufarelli V, Baghban-Kanani P, Azimi-Youvalari S, Hosseintabar-Ghasemabad B, Slozhenkina M, Gorlov I, et al. (2022). Effect of dietary flaxseed meal supplemented with dried tomato and grape pomace on performance traits and antioxidant status of laying hens. Anim Biotechnol.

[24] Jafari M, Pirmohammadi R, Bampidis V (2006). The use of dried tomato pulp in diets of laying hens. Int J Poult Sci.

[25] Nobakht A, Safamehr AR (2007). The effects of inclusion of tomato pomace in laying hens. J Anim Vet Adv.

[26] Yitbarek B (2013). The effect of feeding different levels of dried tomato pomace on the performance of Rhode Island Red (RIR) grower chicks. Int J Livest Prod.

[27] Panaite TD, Nour V, Vlaicu PA, Ropota M, Corbu AR, Saracila M (2019). Flaxseed and dried tomato waste used together in laying hens diet. Arch Anim Nutr.

[28] Mahata ME, Manik J, Taufik M, Rizal Y, Ardi A (2016). Effect of different combinations of unboiled and boiled tomato waste in diet on performance, internal organ development and serum lipid profile of broiler chicken. Int J Poult Sci.

[29] Jouzi H, Vali N, Pourreza J (2015). The effects of tomato pulp powder supplementation on performance and some blood parameters in Japanese quail (*Coturnix japonica*. ARPN J Agricult Biol Sci.

[30] Safamehr A, Malek H, Nobakhat A (2011). The effect of different levels of tomato pomace with or without multi-enzyme on performance and egg traits of laying hens. Iran J Appl Anim Sci.

[31] Habanabashaka M, Sengabo M, Oladunjoye IO (2014). Effect of tomato waste meal on lay performance, egg quality, lipid profile and carotene content of eggs in laying hens. Iran J Appl Anim Sci.

[32] An BK, Choo WD, Kang CW, Lee J, Lee KW (2019). Effects of dietary lycopene or tomato paste on laying performance and serum lipids in laying hens and on malondialdehyde content in egg yolk upon storage. J Poult Sci.

[33] Kim JH, Hong ST, Lee HS, Kimt HJ (2004). Oral administration of pravastatin reduces egg cholesterol but not plasma cholesterol in laying hens. Poult Sci.

[34] Bala DA, Matur E, Ekiz EE, Akyazi I, Ergen E, Erek M, et al. (2020). Can dried tomato and red pepper powder be used as a dietary supplement to strengthen defence systems and production performance in laying hens?. Eur Poult Sci.

[35] Honda M, Ishikawa H, Hayashi Y (2019). Alterations in lycopene concentration and Z-isomer content in egg yolk of hens fed all-E-isomer-rich and Z-isomer-rich lycopene. Anim Sci J.

[36] Kaspers B (2016). An egg a day – the physiology of egg formation. Lohmann Inf.

[37] Mahata ME, Rizal Y, Hermansyah D, Nurhuda GA (2016). Effects of boiled tomato waste utilization in the diet on serum lipid profile and egg quality of laying-hens. Int J Poult Sci.

[38] Wang H, Wu K, Mi X, Rajput SA, Qi D (2023). Effects of 3-hydroxy-3-methylglutaryl-CoA reductase inhibitors on cholesterol metabolism in laying hens. Animals.

[39] Elkin RG, Hester PY, ed. (2016). Cholesterol in chicken eggs: still a dietary concern for some. Egg innovation and strategies for improvement.

[40] Elkin RG, Furumoto EJ, Thomas CR (2003). Assessment of egg nutrient compositional changes and residue in eggs, tissues, and excreta following oral administration of atorvastatin to laying hens. J Agric Food Chem.

